# Recurrent Spinal Canal Stenosis after Decompression and Fusion Surgery Due to Bone Overgrowth: Case Report

**DOI:** 10.3390/medicina60091554

**Published:** 2024-09-23

**Authors:** Yong-Chan Cho, Dae-Woong Kim, Soon-Do Wang, Chang-Hyun Kim, Chang-Hwa Hong

**Affiliations:** Department of Orthopaedic Surgery, Soonchunhyang University Hospital Cheonan, 31, Suncheonhyang 6-gil, Dongam-gu, Cheonan 31151, Republic of Korea; dhsdyd12@naver.com (Y.-C.C.); kkkdw7777@naver.com (D.-W.K.); wangwangwang11@gmail.com (S.-D.W.); s99942@schmc.ac.kr (C.-H.K.)

**Keywords:** pain of lower spine, decompression, spinal canal

## Abstract

Bone regrowth commonly occurs following lumbar decompression surgery. Although it is rare for neurological symptoms to occur due to bone regrowth, this study describes two patients who underwent both decompression and fusion surgeries but experienced spinal canal restenosisthat was severe enough to induce neurological symptoms. A 32-year-old man underwent posterior decompression and posterior lumbar interbody fusion for lumbar canal stenosis at the L4/L5 level 5 years prior. However, the sudden onset of lower back pain occurred 5 years later. Bone overgrowth on the left side was observed at the L4/L5 level. A second surgery was performed, and the patient’s symptoms improved significantly. In the second case, a 56-year-old man underwent posterior decompression and posterior lumbar fusion at the L3/L4 and L4/L5 levels for an L4 burst fracture. One month later, he complained of lower back pain and underwent a second posterior decompression surgery. One year later, he presented to an outpatient clinic with lower back pain and neurological symptoms. Bone overgrowth was observed on both sides at the L4/L5 level. Thus, screw removal and laminectomy at the L3, L4, and L5 levels were performed as a third surgery. This study discusses the factors affecting bone regrowth and the methods that can be used to prevent it.

## 1. Introduction

Decompression and fusion surgeries are commonly used surgical methods for spinal disorders such as herniated intervertebral discs or spinal stenosis, accompanied by neurological symptoms. However, several studies have shown that bone regrowth occurs after such surgeries [1,2,3]. Gradual regrowth of the laminae and articular processes that were partially resected during surgery is common. However, bone regrowth after lumbar decompression and fusion surgery does not lead to neurological symptoms [4]. To reduce the recurrence of restenosis due to bone regrowth, studies have reported that the rate of restenosis is lower when spinal fusion surgery is performed in addition to decompression surgery compared to when decompression surgery is performed alone. However, this report discusses two cases of spinal canal restenosis due to bone overgrowth that led to neurological symptoms despite simultaneous decompression and fusion surgery.

## 2. Case Presentation

### 2.1. Case 1

**Figure 1:**
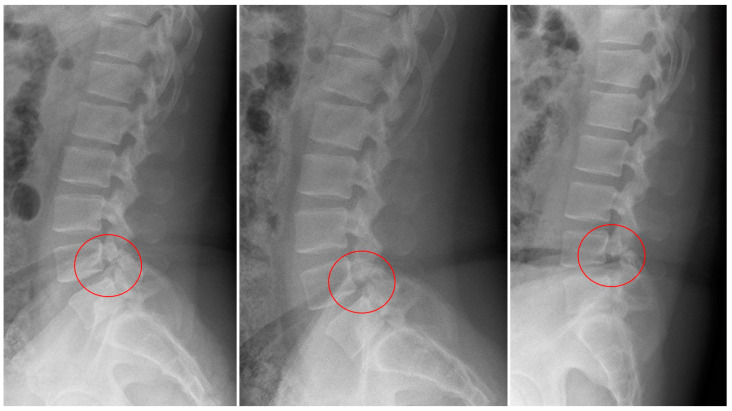
L-spine lateral radiography before surgery shows spondylolisthesis at the L4/L5 level.

**Figure 2:**
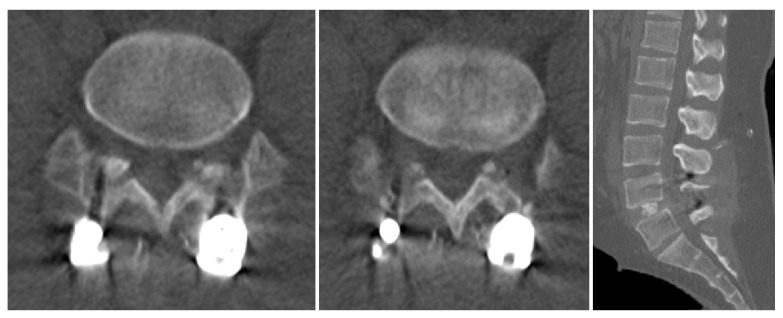
CT image after the aggressive decompression and fusion surgery at L4/L5.

**Figure 3:**
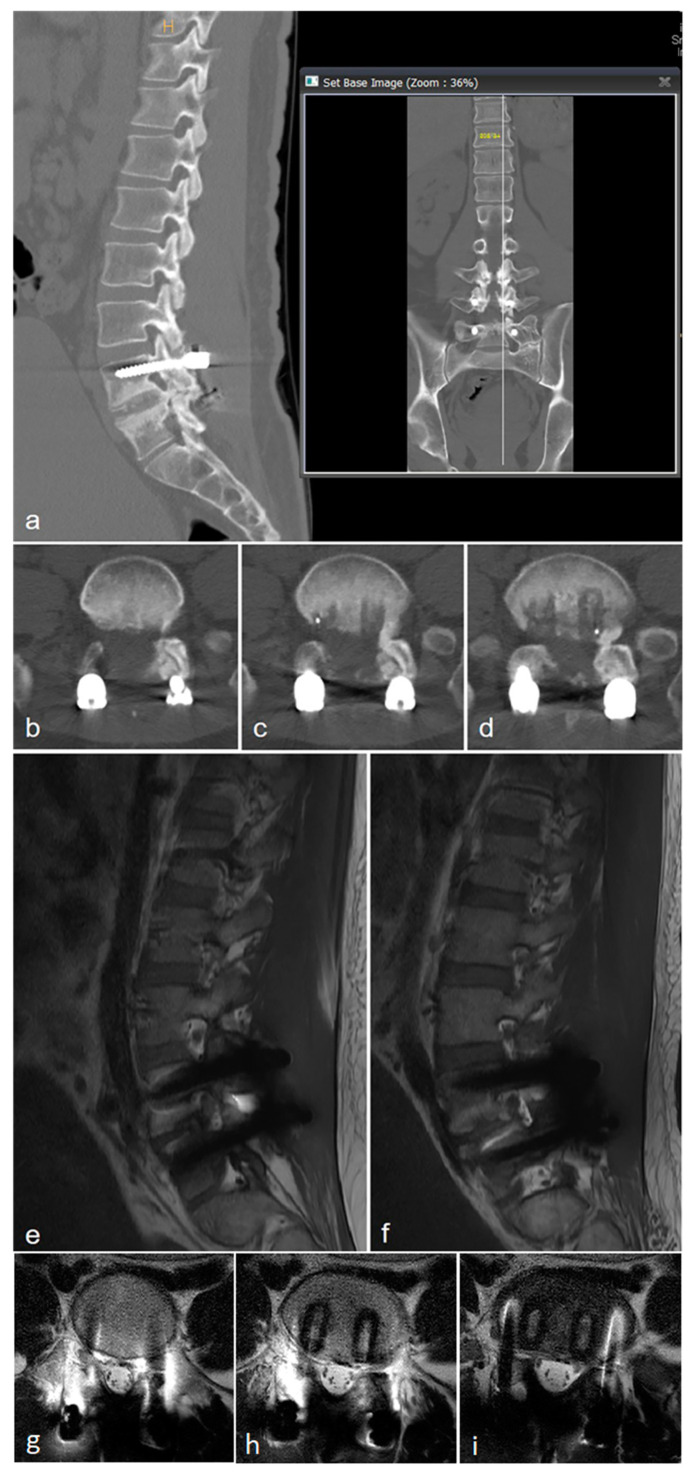
Images 5 years after the surgery. (**a**–**d**) CT image shows restenosis of facet joint on left side at L4/L5 level. (**e**–**i**) L-spine MR image shows restenosis of left side at L4–5 level.

**Figure 4:**
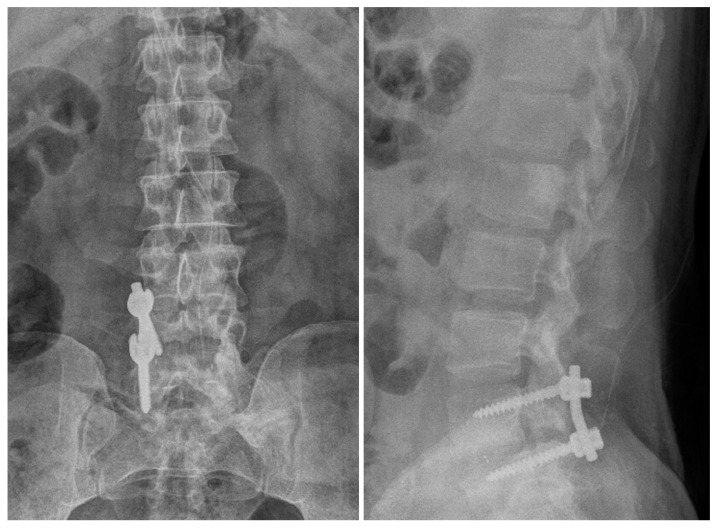
Radiographicimage after the aggressive decompression and fusion surgery at L4/L5.

A 32-year-old male patient presented to the emergency room with severe lower back pain (LBP) radiating to the left lower limb, which occurred suddenly while rising from a seated position. During the physical examination, his motor and sensory functions were deemed to be intact, but the patient experienced significant pain, with a Visual Analog Scale (VAS) score of 8, complaining of difficulty walking. The patient had undergone posterior decompression and posterior lumbar interbody fusion (PD and PLIF) surgery for isthmic spondylolisthesis 5 years prior. Before surgery, severe stenosis on the right side at the L4/L5 level was observed on radiographic examination (Figure 1). Aggressive decompression was performed on the right side at the L4/L5 level (Figure 2). Although the patient’s symptoms improved post-surgery, the patient reported a sudden onset of lumbar pain 5 years after. Restenosis due to bone overgrowth was detected on the left side of the L4/L5 facet joint in computed tomography (CT) (Figure 3a–d). Magnetic resonance imaging (MRI) also showed evidence of bone overgrowth on the left side of L4/L5 (Figure 3e–i). After performing a diagnostic selective nerve root block (SNRB) at the left L4/L5 level, the patient’s symptoms improved, confirming the clinical correlation with restenosis at the L4/L5 level. Considering the patient’s current clinical symptoms, surgery was planned. The fusion status on imaging was found to be stable; therefore, removal of the screws from the left fusion site and posterior decompression were planned as theoperative methods. Intraoperative findings confirmed the presence of bone overgrowth at the L4–5 level, and screw removal and posterior decompression were performed accordingly (Figure 4). Following surgery, the patient’s LBP and radiating pain in the left lower limb improved from a VAS score of 8 to 0. Furthermore, CT imaging performed 1 month and 3 months post-surgery did not reveal any abnormal findings at the facetectomy site, and the patient’s symptoms improved and remained stable.

### 2.2. Case 2

**Figure 5:**
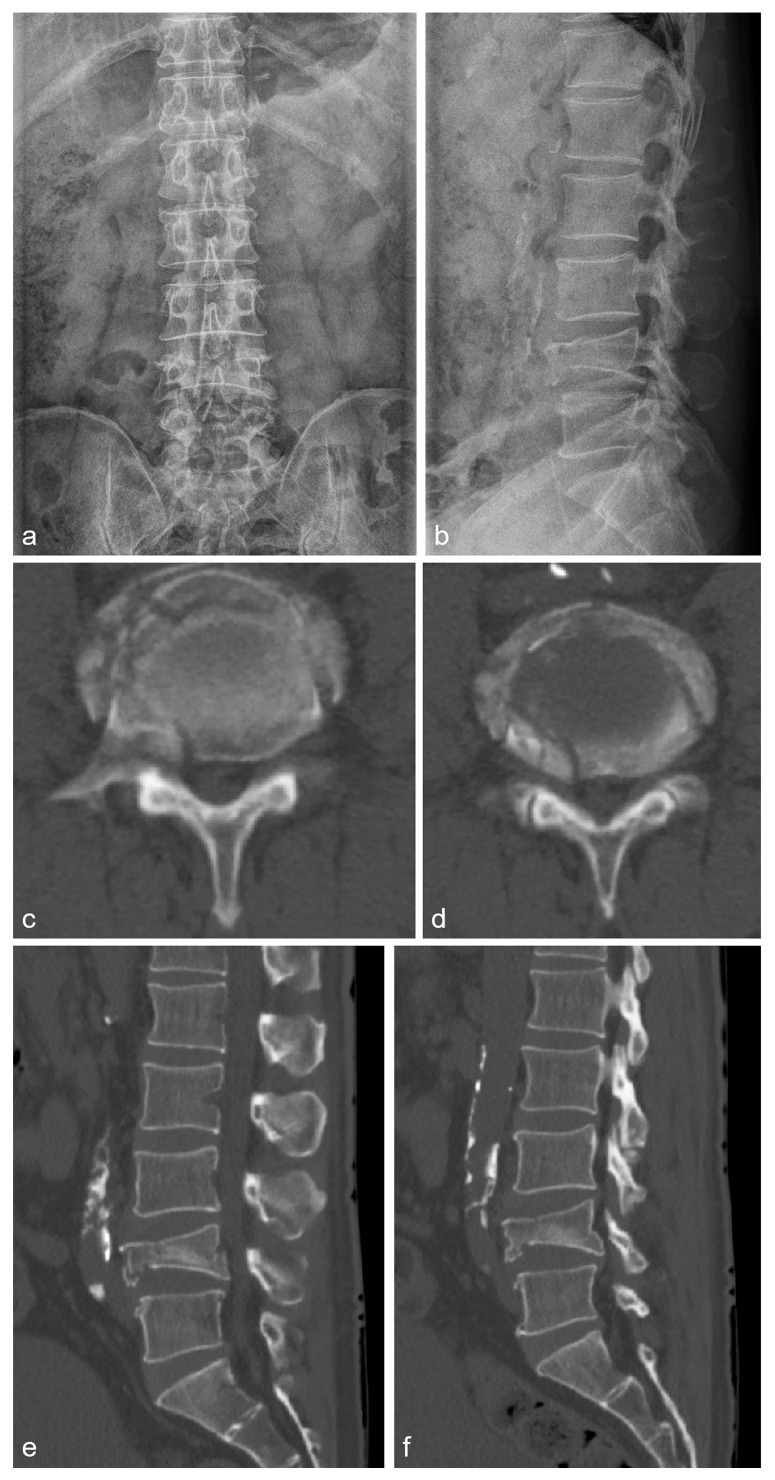
(**a**,**b**) Radiographic image before the first surgery showing burst fracture at L4. (**c**–**f**) CT image before the first surgery showing central canal stenosis at the L4/L5 level.

**Figure 6:**
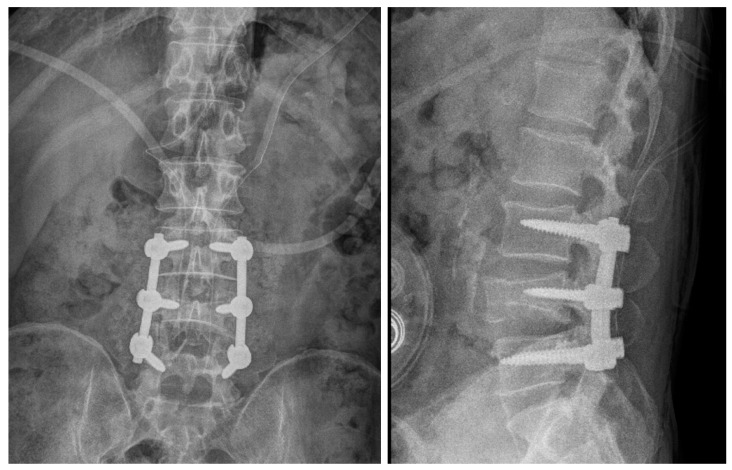
Radiographic image after the first surgery showing L3/L4/L5 fusion state.

**Figure 7:**
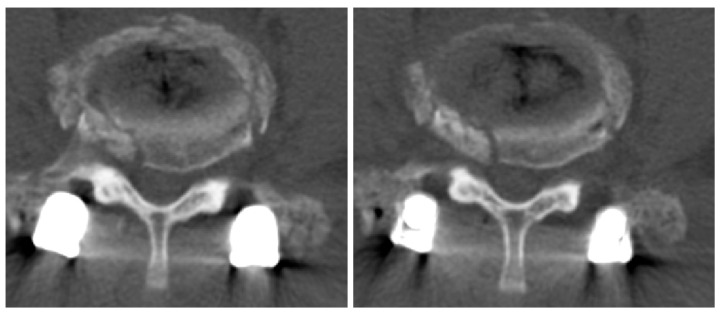
CT image 1 month after first surgery showing post-traumatic stenosis on the right side at the L4/L5 level.

**Figure 8:**
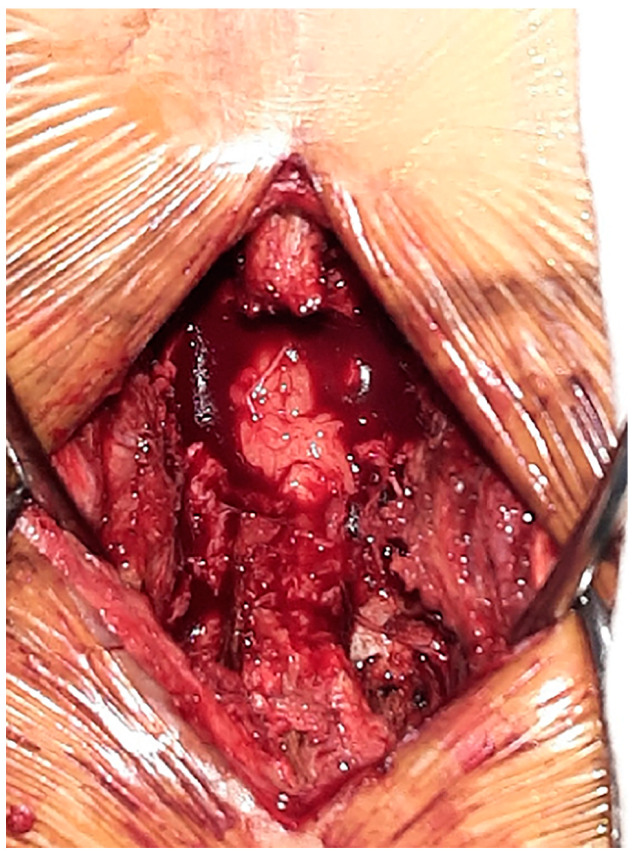
Posterior decompression (facetectomy) surgery was performed.

A 56-year-old male patient presented to the emergency room 2 years prior with severe LBP after an impact with a speed bump while driving a dump truck. Radiographic images showed a burst fracture at the L4 level (Figure 5a,b), as well as central stenosis, predominantly on the left side at the L4/L5 level (Figure 5c–f). The patient underwent posterior decompression and posterior lumbar fusion (PD and PLF) surgery at the L3/L4 and L4/L5 levels (“first surgery”; Figure 6). However, one month post-surgery, the patient experienced radiating pain and numbness in both lower legs during ambulation. CT imaging showed post-traumatic stenosis at the L4/L5 level (Figure 7), which led to lumbar canal restenosis at the L4/L5 level. Subsequently, posterior decompression at the L4/L5 level was performed (“second surgery”; Figure 8). After surgery, the patient’s symptoms improved, and he was discharged.

**Figure 9:**
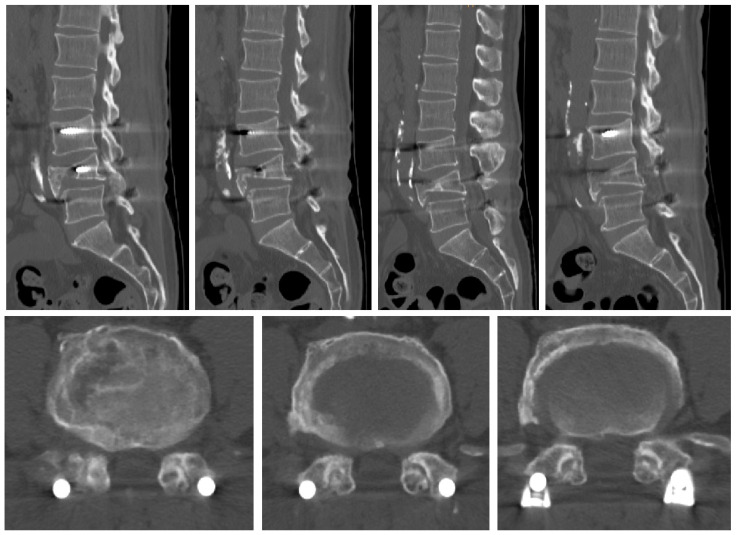
CT imaging showed central stenosis and bilateral neural foraminal stenosis at L4/L5 due to bone overgrowth.

**Figure 10:**
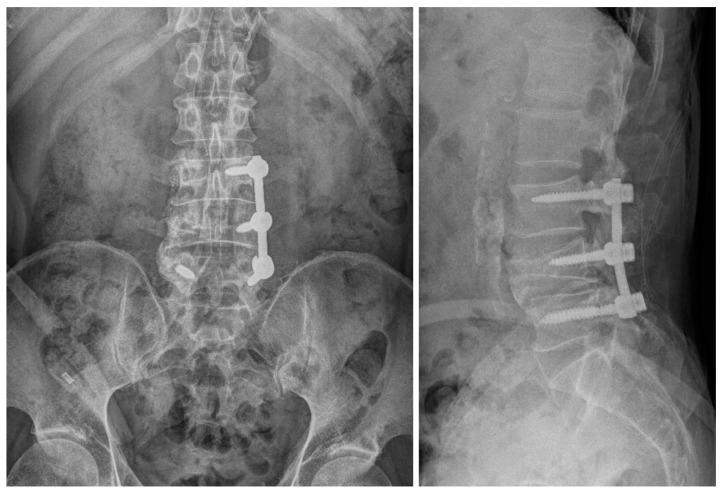
Radiographic images after the third surgery showing a well-decompressed state.

One year after the second surgery, the patient presented to the outpatient clinic after experiencing the sudden onset of LBP with a VAS score of 4 and right buttock pain with a VAS score of 7, along with radiating pain in his right leg. Physical examination revealed reduced motor function in right ankle dorsiflexion (grade IV/V) and diminished sensory perception at the L4 and L5 dermatome levels. CT imaging revealed central stenosis and bilateral neural foraminal stenosis at the L4/L5 level due to bone overgrowth (Figure 9). Considering his stable fusion status on imaging, the patient underwent surgery involving screw removal and laminectomy for posterior decompression at the L3/L4 and L4/L5 levels (“third operation”; Figure 10).

After surgery, the patient’s LBP was resolved, and the radiating pain in his right leg improved from a VAS score of 7 to a VAS score of 2. Additionally, the neurological symptoms in his right lower limb improved, with intact motor and sensory functions. CT imaging performed 3 months after surgery demonstrated an improvement in right foraminal stenosis without any symptomatic recurrence, indicating a stable condition.

## 3. Discussion

Lumbar canal restenosis with neurological symptoms is an exceedingly rare condition that manifests as bone overgrowth after surgery. Bone regrowth at decompression sites has been welldocumented [1,2,3]. Postacchini and Cinotti [1] observed bone regrowth in 88% of their patients following total or bilateral laminectomies. Bone regrowth typically manifests in the laminae and articular processes resected during surgery [1]. Similarly, Chen et al. [2] performed a multivariate analysis of 48 patients and reported that bone regrowth occurred in most of the patients who underwent decompression surgery.

It has been reported that performing fusion surgery along with decompression tends to result in a better prognosis than performing decompression alone [5]. However,Shimauchi-Ohtaki et al. [6] suggested that revision surgery leads to a better prognosis when restenosis of the lumbar canal occurs due to bone overgrowth. Nevertheless, there is no difference in the prognosis between decompression surgery alone and combined decompression and fusion during revision surgery [7].

Dazley et al. [8] found that lumbar restenosis has many etiologies, including the formation of osteophytes due to the motion of the spinal segment, residual ligamentum flava, and epidural scarring. However, some cases may be correlated with lumbar restenosis with inadequate decompression. Dazley et al. [8] highlighted that spinal instability caused by aggressive resection of the facet joints can result in lumbar restenosis. Furthermore, Chen et al. [2] reported that postoperative spinal instability is significantly associated with bone regrowth, whereas the depth of decompression does not affect bone regrowth. They insisted that multilevel fenestration with medial foraminotomy was a better option than wide decompression. However, the mechanisms underlying symptomatic restenosis being caused by postoperative instability remain unclear.

Two distinct types of bone regrowth were identified by Postacchini and Cinotti [1]: (a) gradual regrowth of the laminae and articular processes that were partially resected during surgery and (b) enlargement and merging of the bone tissue islets within the fibrous sheet filling the laminectomy or laminotomy defect. The former process primarily narrows the surgical defect in the transverse plane, while the latter, which occurs subsequent to the former, significantly contributes to lamina regrowth at the surgical defect ends, leading to regrowth of the vertebral arch medially to the posterior joints.

While regrowth of the posterior arch enhances vertebral stability, it may also lead to narrowing of the nerve root canal or the central portion of the spinal canal. Narrowing of the vertebral canal is primarily attributed to the regrowth of the posterior facet joints, which may reproduce the pathological conditions that occurred prior to surgery. Conversely, the regrowth of the laminal arch typically does not cause significant compression, except in cases of degenerative spondylolisthesis. In such instances, regrowth of the laminal arch may contribute to neural structure compression at the level of the intervertebral space, similar to the original laminae pre-surgery.

Previous studies [1,4,5,9] have extensively investigated the treatment methods for spinal canal restenosis caused by bone overgrowth. Postacchini and Cinotti [1] highlighted that instrumented fusion procedures can potentially reduce the risk of recurrent stenosis. Similarly, Cassinelli et al. [5] demonstrated an association between improved fusion rates and the long-term clinical outcomes after instrumented fusion. Herkowitz et al. [4] conducted a comparative study of patients undergoing decompression with and without instrumented fusion and concluded that instrumented fusion yielded superior results without a concurrent increase in complication rates. Despite research [9] suggesting favorable outcomes for restenosis following decompression and fusion surgery, the two cases presented here exhibited severe lumbar canal restenosis accompanied by neurological symptoms despite undergoing decompression and fusion surgery.

Patients diagnosed with degenerative spondylolisthesis who undergo fusion surgery exhibit some degree of bone regrowth, which is generally lower than that in patients without fusion. This indicates that multiple factors may contribute to posterior arch regrowth. However, in these cases, bone regrowth occurred before achieving solid fusion.

Patients with vertebral instability before or after surgery exhibit greater bone regrowth on average compared to those without instability. Conversely, spinal segments with restricted mobility, often owing to extensive vertebral body osteophytes, show less bone regrowth than those with normal mobility. In particular, the L4–L5 segment, known for its high mobility among the lumbar intervertebral levels, exhibited the highest rate of bone regrowth. These observations suggest that the regrowth of the posterior arch might be prompted by abnormal vertebral motion, serving as an effort to enhance vertebral stability.

In the present case, the patients underwent decompression surgery again to treat their restenosis. Mendenhall et al. [7] reported that revision decompression and instrumented fusion are effective in treating lumbar canal restenosis, and some studies [6,8] have reported that the surgical outcomes of patients who undergo decompression alone are similar to those of patients who undergo decompression and fusion surgery for revision.

Numerous studies [1,2,3] have demonstrated the mechanisms underlying bone overgrowth that cause spinal canal restenosis. However, other factors contributing to this clinical outcome remain unclear. Thus, the etiology of spinal canal restenosis and methods for preventing it should be studied comprehensively.

## 4. Conclusions

Recurrent spinal canal stenosis due to bone overgrowth after decompression and fusion surgery poses significant challenges for clinical management. Patients commonly experience bone regrowth in surgical defects as part of the natural postoperative process. Therefore, when deciding on a surgical approach, it is essential to consider reducing the vertebral instability to prevent bone regrowth. Through comprehensive evaluation, timely diagnosis, and appropriate surgical intervention, favorable outcomes can be achieved in patients presenting with recurrent symptoms. Continued research and clinical vigilance are imperative for advancing our understanding and management of this complex condition.

## Data Availability

The datasets used and/or analyzed during the current study are available from the corresponding author upon reasonable request.
